# Leishmaniasis patients' pilgrimage to access health care in rural Bolivia: a qualitative study using human rights to health approach

**DOI:** 10.1186/s12914-019-0196-4

**Published:** 2019-03-05

**Authors:** Daniel Eid, Miguel San Sebastian, Anna-Karin Hurtig, Isabel Goicolea

**Affiliations:** 1 Department of Biomedical Sciences Research, Faculty of Medicine, San Simon University, Aniceto Arce Avenue, 371 Cochabamba, Bolivia; 2 Department of Public Health and Clinical Medicine, Epidemiology and Global Health, Umeå University, SE-901-85 Umeå, Sweden

**Keywords:** Cutaneous leishmaniasis, Human rights, Health care, Seeking behavior, Bolivia

## Abstract

**Background:**

Leishmaniasis is a neglected tropical disease endemic in Bolivia that disproportionately affects people with little social and political capital. Although the treatment is provided free of charge by the Bolivian government, there is an under-utilization of treatments in relation to the estimated affected population. This study explores the experiences of patients with leishmaniasis and the challenges faced when searching for diagnosis and treatment in Bolivia using a human rights approach.

**Methods:**

We conducted open-ended interviews with 14 participants diagnosed with leishmaniasis. The qualitative data were analysed using thematic analysis and were interpreted under a human rights approach to health care.

**Results:**

Four themes emerged during data analysis: (1) the decision for seeking a cure takes time; (2) the severity of symptoms and disruption of functioning drives the search for Western medicine; (3) the therapeutic journey between Western and traditional medicine; and (4) accessibility barriers to receive adequate medical treatment. This study showed that access to health care limitations were the most important factors that prevented patients from receiving timely diagnosis and treatment. Cultural factors played a secondary role in their decision to seek medical care.

**Conclusions:**

Accessibility barriers resulted in a large pilgrimage between public health care and traditional medicinal treatments for patients with leishmaniasis. This pilgrimage and the related costs are important factors that determine the decision to seek health care. This study contributes to the understanding of the under-utilisation problems of medical services in leishmaniasis and other similar diseases in remote and poor populations.

**Electronic supplementary material:**

The online version of this article (10.1186/s12914-019-0196-4) contains supplementary material, which is available to authorized users.

## Background

Neglected tropical diseases (NTDs) are a group of diseases that affect almost exclusively poor people living in rural parts of low-income countries, and their most important common feature is that they affect disproportionately individuals with little social and political capital [1]. In this context, a human rights approach is key to strength health advocacy for the people affected by NTDs and to support control and elimination efforts of these diseases [2].

NTDs are both a cause and consequence of human rights violations. NTDs are more likely to occur where human rights are not guaranteed and they may lead violations of human rights and fundamental freedoms, including equality and non-discrimination [3].

The right to health defined as “the highest attainable standard of health”, is a legally binding obligation to states that are party to any international covenant that contains it, and obliges those states to advance and secure it, whether through domestic legislation, policies or otherwise. It is a progressive right that states committed to its protection must secure over time. Several international human rights treaties, as well as regional agreements and national constitutions and laws, protect the right to health [4–8]. The main global treaty that enshrines this right is the International Covenant on Economic, Social and Cultural Rights (ICESCR) [8]. The committee of the ICESCR outlines four essential conditions of health systems necessary for its realization: availability, accessibility, acceptability and quality, also known as the AAAQ framework [9, 10]. The World Health Organization (WHO) suggests NTDs program managers and partners to monitor differences in access using this framework to explore barriers and facilitating factors for effective coverage and to understand who is left behind and why [11].

Leishmaniasis is considered one of the most NTDs due to lack of funding, political commitment and national and international cooperation [12]. WHO estimates 0.7–1 million annual cases worldwide due to tegumentary leishmaniasis [13] while in 2016 in Latin America,, 48,915 new cases were reported [14]. Tegumentary leishmaniasis has several clinical forms ranging from cutaneous leishmaniasis (CL) to mucosal leishmaniasis (ML). CL exists mostly as painless skin ulcers that can take months to years to heal [12] and ML usually appears after a CL episode and produces severe destruction in the mouth, nose and larynx if not treated [15]. CL affects patients’ quality of life in addition to affecting work and school activities [16]. CL scars produce loss of self-esteem, depression, anxiety and stigma and it is an obstacle to the social integration of those affected by the disease [17]. This situation may be even worse in ML due to the disfigurement and mutilation of facial structures. In the most severe cases of ML, there might be an affectation of speech ability as well as vital functions such as breathing and feeding.

Bolivia is in the top five countries in South America with leishmaniasis cases and is considered the country with the highest risk of ML [18]. Seven of the nine departments located in the Amazon rainforest are considered endemic and four of them (La Paz, Pando, Beni and Cochabamba) contain most of the reported cases nationally [19].

Leishmaniasis is a growing problem in Bolivia. In the last 33 years, 52,244 cases of leishmaniasis have been recorded, of which 92% were CL and 8% ML [19]. To reduce the morbidity and mortality of leishmaniasis, the National Leishmaniasis Control Program (NLCP) was created in 2007 with the aim of improving the access and quality of health care in terms of prevention, diagnosis and treatment. However, it is well known that a high number of people cannot access adequate health care to treat this disease.

Although some studies have discussed the difficulties in access to health care for patients with leishmaniasis in Latin America [20–23], these are few, old, do not focus specifically on the problem of access to medical care and none of them has been conducted in Bolivia.

The purpose of the present study is to explore the experiences of patients with leishmaniasis and the challenges they faced when searching for diagnosis and treatment in the Amazon region of Bolivia using a human rights to health approach.

## Methods

### Study area and population

Bolivia is a country located in South America with a population of over 10 million people. It has more than 36 ethnic groups, and one-third of the population lives in rural areas [24]. The percentage of health expenditures of the national gross domestic product (GDP) was 6.3%, and the number of physicians per 10,000 population was 14.8 in 2016 [25].

The Bolivian health system has been acknowledged by the World Health Organisation as pluralistic, meaning that different types of medical care coexist: (i) the traditional medicine system, which includes the care provided by the patients themselves, traditional healers and herbalists; and (ii) the Western medicine system, which is a biomedically based system that includes medical care provided in both the public and private health sectors [26].

Because the main mechanism of transmission of leishmaniasis in the country is sylvatic, meaning that the rainforest is the ecosystem of vectors and reservoirs, there are few options to control vector exposure. For this reason, control strategies are focused mainly on secondary prevention, which is effective diagnosis and treatment. NLPC provides free treatment based on antimonial drugs (meglumine antimoniate for 20 days via intramuscular injection in a medical centre). Confirmation of the presence of the parasite in the lesion by the laboratory is a prerequisite for treatment.

### Participants

In total, 14 participants (11 male and 3 female) between the ages of 17–50 years were recruited. Participants were from various backgrounds, several geographic settings and had different types of problems related to leishmaniasis. There was one case of CL in a one-year-old child where we interviewed the mother (Table 1).

**Table 1:** Participants´ sociodemographic characteristics

Sociodemographic characteristics		Count
Ethnic Group	Mestizo	8
	Tsimane	6
Age	Below 30 years old	3
	From 30 to 49 years old	9
	Older than 50 years old	2
Place of Residence	Cochabamba	5
	Beni	6
	La Paz	1
	Pando	1
	Potosi	1
Occupation	Housewife	2
	Student	2
	Farmer	9
	Mechanic	1
Form of leishmaniasis	Cutaneous Leishmaniasis	10
	Cutaneous Leishmaniasis with therapeutic Failure	2
	Mucosal Leishmaniasis	2

All participants lived in endemic rural areas and had experienced leishmaniasis. The first group of eight participants was contacted by patients who visited the University Centre of Tropical Medicine (CUMETROP) in Cochabamba seeking medical attention for leishmaniasis and during leishmaniasis medical campaigns in their communities. CUMETROP is a research institute on tropical infectious diseases specialising in the diagnosis and treatment of leishmaniasis. CUMETROP's reputation in treating leishmaniasis attracts patients from different regions of the country. The second group of six participants from the Tsimane communities were contacted through the Tsimane Health and Life History Project's medical brigade that provides medical attention to these communities. The Tsimane is an Amazonian indigenous group of around 10,000 people who are settled deep in the forest and their livelihood depends mainly on hunting and fishing [27]. Because of the lack of adequate roads, public transport is scarce. Consequently, their ability to access to medical services is limited.

### Data collection

The interviews were performed by the first author, a Bolivian physician trained on qualitative methods. In Cochabamba, interviews were conducted in a private and comfortable space located in the medical office of CUMETROP and in the community health care centres. In the Tsimane communities, interviews were conducted in a private space close to the settlement of the medical brigade. Each in-depth interview lasted between 30 and 45 min. At the beginning of the interview, the interviewer explained the general topic of the interview and encouraged the interviewee to express ideas freely. The interview guide (Additional file 1) included semi-structured open-ended questions with some key topics: (1) perception of leishmaniasis threat; (2) coping response to leishmaniasis; (3) experiences with traditional and Western medicine; (4) economic, social, emotional and productive consequences of leishmaniasis.

The interviews were conducted mainly in Spanish, the mother tongue of the interviewer. For the interviews with Tsimane participants, interpreters were used. All the interviews were digitally recorded and transcribed verbatim.

### Data analysis

Interviews were analysed thematically [28, 29]. All authors read the transcripts. A preliminary data analysis was performed with two interviews to look for recurring concepts that were codified. QSR International’s NVivo 10 software was used to index material and for retrieval of text chunks that corresponded to similar codes. The codes with similar information were arranged and collated into groups, and a code list was developed. DE did the initial coding, and these initial codes were discussed with IG. Afterwards, the other 12 interviews were analysed using the code list as a guide. The analysis of these 12 interviews allowed us to refine the code list and collate codes with similar meaning into themes. DE and IG grouped the codes into five preliminary themes that were discussed with MSS and AKH. After several rounds of discussions and refinements within the team and a process of continuously getting back to the transcripts, the five preliminary themes were condensed into the four themes presented herein.

## Results

Four themes were identified that reflect the pilgrimage of patients with leishmaniasis from the appearance of the lesion until they could receive appropriate treatment: (1) the decision for seeking a cure takes time; (2) the severity of symptoms and disruption of functioning drives the search for Western medicine; (3) the therapeutic journey between Western and traditional medicine; and (4) accessibility barriers to receive adequate medical treatment.

### The decision for seeking a cure takes time

At the beginning of the illness, the CL wound was considered a minor problem that did not deserve attention. All participants reported that they thought the wounds were common problems related to farming activities and daily life experiences in the forest. Common comments mentioned by some participants were: “*I thought it was a mange,*” *“It was just a little pimple,” “I thought it was a thorn nailed in my leg.”* Participants agreed that the first reaction was to wait for spontaneous healing as one participant commented:

*“It was a simple little thing. It was very small. I thought it would heal over time because the ‘carachitas’ (manges) heal by themselves. Isn’t it?” (Informant 12, mestizo man with CL).*

The participants emphasised the fact that the wound was painless as a reason for their delay in seeking attention. Others also noted that it was not a problem as long as it did not affect their ability to work. One participant commented:

*“It was not a problem for me. The wound did not hurt. I could continue working without problems.” (Informant 4, mestizo man with CL).*

Some participants preferred to wait a long time before starting to worry about healing the wound. These waiting times were up to 6 months and even one year in some cases. Others described very severe complications before deciding to seek a cure.

*“... I was busy with my work, so I did not pay attention to my wound. While the days passed working, the wound continued growing, little by little, deeper and it had made big and deep. Then, almost a month later, I realised that it had become too deep. I could insert part of my finger into the hollow of the wound. At that moment, I was scared and I went to the health post.” (Informant 12, mestizo man with CL).*

However, the long healing time of the wound led people to inquire among their social networks about the problem. All participants mentioned having a relative or acquaintance who knew about the disease. The disease was known as “espundia” among mestizos and “jadyeye’” among the Tsimane. The majority of the mestizo participants recounted visiting the health post closest to the community or private doctors.

*“My husband had the same problem 5 years ago. The wound had the same structure. So, I decided to take my son directly to the medical post.” (Informant 11, mestizo mother of a child with CL).*

*“I applied ají (chilli pepper) to my wound one full day. When I took it out the next day nothing had happened. Then my daughter told me to seek help to the medical post.” (Informant 5, Tsimane woman with CL).*

For Tsimane, traditional medicine was their only option due to the unavailability of health care centres in their area. They described using medicinal plants to heal the wound in addition to a series of practices to prevent the growth of the ulcer. For instance, medicinal plants such as japainiqui, tobacco and pepper were applied as poultices in the wound. The healing time of the wound with these treatments was between four and six months. One participant also commented on his experience with the direct application of fire in the wound.

*“When I had the disease, my grandmother used fire to treat it. She put fire directly on the wound of my face. I was a child, and it hurt me a lot. She also used a poultice with japainiqui (traditional plant). Additionally, she forbade me to eat food with salt and oil.” (Informant* 9*, Tsimane indigenous man with CL).*

In addition to the use of medicinal plants, participants described a series of practices related to diet and their daily activities to prevent ulcer growth. One participant shared his experience:

*“My wife used to tell me that I could not touch her because the wound could grow. We could not have sex. In addition, I had to take care to avoid water contact with the wound. I could not eat with salt or oil neither.” (Informant 10, Tsimane indigenous man with CL).*

### The severity of symptoms and disruption of functioning drives the search for Western medicine

A key determining factor often mentioned in the decision to use Western medicine was the severity of symptoms and disruption of functioning. Concern about the worsening of the disease, affecting their leisure and work activities, and in the most serious cases, interference with necessary habits such as sleeping, or eating were stated as the main factors affecting their life.

The experience of one of the Tsimane participants illustrates this point. The concern produced by the fact that the wound was not healing but actually worsening after several attempts with traditional medicine led him to make a journey of more than one day of walking in search of medical care.

*“I used japainiqui and other plants, but it didn’t heal. Then, somebody told me there was treatment in the parish of San Borja. So, I told my wife that I would travel to receive treatment, and I did it.” (Informant 8, Tsimane indigenous man with CL).*

A similar experience was experienced by a mestizo participant who preferred traditional medicine as the first treatment. He described his attempts to treat the lesion with the use of poultices that produced infections that affected his work and leisure activities:

*“I put tobacco on it, and the wound became very infected. My leg was very swollen. I put more tobacco on it and the wound filled with more pus. I also noticed that the wound was growing. It was getting worse. At that moment, I stopped using medicinal plants and I went to the hospital.” (Informant 1, mestizo man with CL).*

The experiences of the participants with ML were even more severe. They described discomfort that affected their ability to talk, sleep and even eat. The great impact on their quality of life led them to seek Western medicine faster.

*“Mucosal leishmaniasis is very severe. I started to cough during the nights, and I couldn’t sleep well... I couldn’t eat. If I ate something that was a bit hot, it hurts me a lot! The pain got to the roots of my teeth. I was really worried. I already visited many hospitals looking for a cure.” (Informant 14, mestizo man with ML)*

### Therapeutic journey between Western and traditional medicine

Often, the first contact of participants with Western medicine in the health centres failed to solve the problem, which subsequently generated mistrust. In some cases, the disease could not be treated due to the lack of a laboratory diagnosis or medication, and patients were referred to specialised centres in the cities:

*“First, I went to a private doctor in Shinaota community who gave me an injection that did not help me in anything. He told me I had leishmaniasis and that I had to save money to make me heal. He sent me to Villa Tunari, but when I arrived, they told me that they could not attend me and that I had to go to Cochabamba city.*” *(Informant 2, mestizo man with CL).*

Another reason for the Western medicine failure was that health posts and the medical staff have no alternative options when conventional treatment fails. A mestizo participant with CL who had a problem of therapeutic failure described his experience.

*“I noticed that it had already passed time after the treatment was finished, but the wound did not heal completely. I went to the doctors to ask for treatment, but they told me to not worry because I had already finished my treatment, and the wound would be healed without doing anything else.” (Informant 12, mestizo man with CL).*

ML appears many years after a CL episode in people who have already left the endemic area and returned to their place of origin. Some patients stated that they visited several health centres where doctors did not know about the disease. They commented that they had gone through several tests and treatments for other diseases without reaching any result or improvement in their disease.

*“I visited many hospitals. They ran many tests on me, but everything was negative. They believed I had cancer. It is cancer. It is cancer, they said, but the results were negative as well. They thought it was tuberculosis, but that was negative too...” (Informant 13, mestizo man with ML).*

In the context of these limitations at the primary health care centres, several mestizo participants described having turned their attention to medicinal plants and traditional healers. However, these treatments were equally ineffective and even harmful.

*“I put on my wound one remedy that the traditional healer sold me. I don’t know what it was. It produced to me an extreme pain. I couldn’t resist it. It was awful...It was like an acid. It swelled my leg. That is why I didn’t use it anymore...” (Informant 12, mestizo man with CL).*

Similar experiences were recounted by patients with ML that required specific treatment to be healed. For these cases, traditional medicine was expensive, and it could not heal the wound.

*“After several attempts with hospitals, I didn’t go anymore. I waited almost a year for it to heal itself, but it was getting worse. So, I went to a traditional healer. He put ant’s poop on my neck and gave me herbal tea to drink. I spent more than 600 BOB (100 USD) on those treatments. It made me feel a little better, but it didn’t cure me.” (Informant 14, mestizo man with ML).*

Other people’s experiences also influenced the decision to choose traditional medicine. One mestizo participant preferred the use of medicinal plants instead of visiting health centres due to previous adverse reactions to leishmanial drugs by his father.

*“My dad also had espundia but he could not complete the treatment. So many injections made him sick. He said that medication made him feel weak and gave him headaches. When my wound appeared, my dad knew that it was espundia. He recommended me that I only should use tobacco leaves because it healed faster and that was better than getting injections.” (Informant 1, mestizo man with CL)*

### Accessibility barriers to receive adequate medical treatment

Although some participants eventually found adequate medical treatment in the specialised centres of the cities, they faced many hardships reaching them.

The long distance was one of the most important barriers with journeys of several days. Poor road conditions and unsafe means of transportation were additional problems to overcome.

*“I have to travel from Yunga communitys in La Paz to Cochabamba (380 km) to receive the medical attention. It is very far, expensive and dangerous. I’ve already had one traffic accident... In my community there are many people with the same disease, but they can’t pay the travel.” (Informant 11, mestizo mother of a child with CL).*

The high economic costs associated with travel were another important difficulty. Some participants reported spending between 500 and 600 USD on transportation, food and lodging. In addition, the lost work time also meant an extra economic burden to the family of the participants. A mestizo participant reflected on his experience:

*“The doctor told me that I need to stay 28 days to receive the treatment. I see it as something impossible. It is a long time without working. Who is going to work for me? If I am not working, how can I get the money to live?” (Informant 12, mestizo man with CL).*

## Discussion

Barriers to accessing health care were the most important factor that prevented leishmaniasis patients from receiving a timely diagnosis and treatment. Cultural factors, such as the use of traditional medicine, played a secondary role. This was an alternative response when leishmaniasis patients failed to find a solution with Western medicine or when this was unavailable. The Bolivian public health system organisation becomes a trap for people from remote environments that have leishmaniasis. Patients with leishmaniasis have to go through a long pilgrimage of health care services and traditional medicine treatments looking for a cure. Even though there is a health post in most of the endemic communities, the conventional treatment and the equipment for laboratory diagnosis are often unavailable. The inability of primary care centres in remote endemic areas to manage diseases has been shown to be a common problem in several Latin American countries with diseases such as Chagas [30, 31], malaria and leishmaniasis [20–23, 32, 33].

From a human rights approach, it is important to remark that health systems are not only structures where technically specialised services are provided, but they must also be considered as key social institutions to ensure the respect for the human right to health and health care [34]. Regarding leishmaniasis, the Bolivian government created the NLCP as a health system response to the disease providing specific medication free of charge for patients. However, there are still several health system factors that prevent patients from fulfilling their right to health. These factors can be organised under the dimensions of the right to health care framework.

### Availability

A human right to health approach requires health facilities, goods and services in sufficient quantity. The World Health Organisation expert committee for leishmaniasis control suggests laboratory confirmation of the parasite in order to receive the treatment given the toxicity risk related to the treatment [35]. For this reason, in Bolivia, the NLCP demands confirmation of the parasite by laboratory diagnosis before delivering the drugs to patients [19]. This recommendation has been shown to be an important obstacle given the Bolivian health system weaknesses. Although in many areas of the country health centres exist at the community level, their conditions are often quite precarious. These services are not equipped with laboratories and do not have medicines for leishmaniasis available. Health facilities that have laboratory services for leishmaniasis confirmation are located in large urban centres far from the sylvatic areas where leishmaniasis occurs. Furthermore, drugs are only available at the NLCP departmental offices in capital cities. Similar findings have been described in Colombia [32] and Ecuador [23], where understaffed medical facilities and lack of medications were the main impediments in receiving adequate treatment. Among Tsimane and other indigenous groups from the Amazon region of Bolivia and Peru [36], the absence of health services resulted in a lack of trust and familiarity with Western medicine as well as more use of traditional medicine.

### Accessibility

The accessibility dimension in the right to health approach has four components: information, physical, economic and non-discrimination [8]. In this study, physical and economic inaccessibility were the most highlighted. Not surprisingly, physical inaccessibility was greatest among indigenous groups. In the Tsimane area, the nearest hospital is located farther than one day of travel from most of the communities. In our study, physical accessibility limitations prevented participants from using Western medicine. One study performed among the Tsimane showed that the communities located farthest from the main town where the regional hospital is located had higher mortality rates across the life course, reflecting the severe consequences of the physical inaccessibility to health care [37]. Additionally, in our study, physical accessibility limitations led participants to use traditional medicine. Similar findings were described in one study performed in Bolivia on indigenous populations of the Amazon rainforest. This study showed that the use and knowledge of medicinal plants were correlated negatively with the distance to the primary health centre [36]. In our study, traditional medicine was very rich in practices and knowledge, mostly with the Tsimane, and included several options that an affected patient could try. These options included diet and practices to avoid the growth of the wound until the use of medicinal plants and chemical substances facilitated its cicatrisation [21–23, 32]. However, traditional remedies have not been demonstrated as effective treatments, and in many instances, can be quite dangerous, as exemplified by our study and other studies in Colombia [32], Guatemala [20], Suriname [21] and Ecuador [23]. These treatments increased the risk of superinfections, scars (worse than those produced by CL), systemic toxicity and carcinogenesis.

Economic accessibility was a big problem as well in patients with leishmaniasis. Even in countries like Bolivia with health care services that are free of charge according to their health policies, there is still a large amount of out-of-pocket expenses. When seeking diagnostic laboratories, patients incur costs, such as transportation, lodging and food, which are quite expensive for patients. In our study, participants described expenditures of up to 600 USD, which can correspond to more than two times the monthly minimum wage in the country. Similar results were found in Suriname and Paraguay where costs equated to one months' salary, with the additional damage of not being able to work during that time and the risk of losing their job [21, 22].

The expensive costs of the illness and the process of seeking health care can seriously affect the household economy of the patients. Similar findings were found in Nepal, Bangladesh, India and Sudan, where many families had to sell part of their livestock or take loans to pay these costs, resulting in catastrophic economic consequences [38–41].

### Quality

An effective health care system with high-quality leishmaniasis diagnosis is a key element in a human rights approach. The factor behind the low-quality problem that emerged in this study was the lack of experience of some physicians in the recognition of the disease. In some cases, this resulted in inadequate and failed treatments, unnecessary examinations and eventually resulted in the loss of confidence in the health system and Western medicine by participants. There are no similar studies in South America that address quality of care for patients with leishmaniasis. However, one study in Colombia mentioned that the participants attributed the scarce use of medical services to the low trust in the quality of primary care services located in their communities [32].

### Acceptability

Although in some Bolivian studies related to medical conditions were different than leishmaniasis, the existence of mistreatment by medical staff or a lack of communication by the medical staff has been described [26, 42, 43]. In our study, no similar complaints were mentioned by the participants.

## Methodological considerations

### Measures to ensure the trustworthiness of the study

The trustworthiness of qualitative research depends on several factors: 1) credibility related to capturing the research questions adequately, 2) dependability related to adequately consider the changes in the research: 3) confirmability when interpretation grounds in the data, and 4) transferability related to the applicability of the findings to other settings [44].

Several measures were taken in the present study to ensure trustworthiness. First, the first author became familiarised with leishmaniasis patients' experiences related to the disease and health services one year before the beginning of the project during medical campaigns performed in communities located in endemic areas [45]. Second, the sample included a wide range of informants from different places in the country and diverse ethnic backgrounds to capture a wide array of experiences [46]. Finally, the interview topics were tested before the study had started on some patients with leishmaniasis to evaluate if the questions were well understood and if they captured the research aim adequately. To strengthen the transferability of this study, a description of the context of the study has been provided, and to enhance dependability, the study adopted an emergent design throughout the research process which contributed to making leishmaniasis patients' voices more visible. Finally, to enhance confirmability, triangulation of researchers from different disciplines and different levels of familiarity with the disease, the study area and the population was used [47]. Two of the authors were more involved in the different stages of designing the study protocol and analysing the data (DE and IG). This allowed the combination of a cultural insider perspective (DE) together with an outsider perspective (IG). In addition, quotations were used to allow the readers assess that the results were grounded on patients’ experiences.

### Limitations and strengths

Two important limitations to consider are the selection process and the translation limitations. Regarding the selection process, the participants in the sample were skewed towards men with a small representation of women, although this also reflects the higher prevalence of leishmaniasis reported among the male population. Regarding translation, it is possible that information could be lost during translation of interviews performed in the native language of Tsimane participants.

As strengths, we can consider two: first, the diversity of the sample that included participants from different places in the country and represented various forms of the illness provided a wider variety of experiences. Second, the information collected from the Tsimane people was relevant, given that it is an isolated and hard-to-reach ethnic group whose experiences are seldom present in research findings. Its conditions and experiences can be used as a mirror of the situation of the other 32 ethnic groups that also belong to the marginalised minorities in the rainforest of Bolivia and maybe elsewhere.

## Conclusions

This study has shown the pilgrimage and difficulties that leishmaniasis patients have to endure when they decide to seek a cure. It shows how this pilgrimage is the product of the limitations and deficiencies of a centralised health system for a disease that disproportionately affects poor populations in remote areas. Unavailability, economic and physical accessibility, and quality of services are important factors that must be addressed in order to allow the exercise of rights on health among patients with leishmaniasis.

Public health initiatives to control leishmaniasis would benefit from using the human rights to health approach and integrating human rights principles; uncovering health inequities and catalyzing integration focusing on who is left behind and why, such that the needs of the most marginalized are met.

Finally, we suggest to policy makers strengthen leishmaniasis care capacity related to laboratory diagnosis and availability of treatments in primary health care centres located in endemic areas.

## Additional file

Additional file 1: In-Depth Interview Guide. (DOCX 26 kb)

## Abbreviations

CL: Cutaneous leishmaniasis
ICESCR: International Covenant on Economic, Social and Cultural Rights
ML: Mucosal leishmaniasis
NLCP: National Leishmaniasis Control Program
