# Supplementary material for: Leishmaniasis patients' pilgrimage to access health care in rural Bolivia: a qualitative study using human rights to health approach
Source: BMC Int Health Hum Rights. 2019 Mar 5;19:12. doi: 10.1186/s12914-019-0196-4 (PMC6402110; doi:10.1186/s12914-019-0196-4)
Supplement: Supplementary file 1 — In-Depth Interview Guide. (DOCX 26 kb) [file 12914_2019_196_MOESM1_ESM.docx]

**In-Depth Interview Guide**

**Date:**

Participant Study ID: ___________

*(Circle one in each row)*

Gender: M F

Type of leishmaniasis : CL ML

Ethnic Group: Tsimane Mestizo Caucasian

Place of Residence:………………..

Ocupation: ………………………………………………….

Age: ……………………..

*Notes to interviewer:*

- *It is not necessary to ask every single prompt. Choose from among the prompts if the information is not forthcoming.*
- *The order of the interview can be modified, according to topics raised by the participant. You can go back to a previous topic or go to a future topic, follow the conversation.*
- *Please tell the intereviewee that we want to learn from him or her, and we would like to hear their experiences; there are no right or wrong answers.*
- *Clearly explain the study purpose to the participant and obtain consent before starting interview.*
- *Write notes after the interview on the interview environment (for example, interruptions, others being present, disturbances, any issues that arose). Do the transcript from the audio recording as soon as possible.*

**Qualitative Field Guide Questions for Wheelchair Users**

1. Please tell me about how did you recognize you have leishmaniasis.

- What did you feel?
- How did you noticed that leishmaniasis would not heal by itself?

1. Tell me about your **first** reactions to the disease.

*Prompts*:

- Who was the person that you visited first looking for an advice?
- Why this person?
- What did he suggest to you?
- Were the advices useful for you?

1. Tell me about your experiences with traditional medicine.

*Prompts*:

- Did you look for natural healers, herbalists or any other traditional healer?
- What did they said to you?
- Did their advices work for you?
- How much did the visits cost to these people?

1. Tell me about your experiences with health care services (western medicine).

*Prompts*:

- What move you to decide to go seeking for medical care?
- How difficult what the transportation to the health service?
- What do they offer to you? Information, laboratory analysis, treatment?
- How did you feel with them?
- Did you need to look for help in any other health care center?
- Did you have to return to the health center? Why?
- How much did cost to you the visit to the health center?
- How did you appraise the attention received?

1. Tell me about how you feel right now

*Prompts*:

- *Are you still receiving some treatment*?
- *Do you need to continue visiting the health care center*?

**Finishing the interview**

Please tell me if you have something else to say that I didn´t ask you. Something you want to add to your answers.

Thanks the informant.
